# MASI, a Smartphone App to Improve Treatment Adherence Among South African Adolescents and Young Adults With HIV: Protocol for a Pilot Randomized Controlled Trial

**DOI:** 10.2196/47137

**Published:** 2023-09-19

**Authors:** Marta I Mulawa, Jacqueline Hoare, Elizabeth T Knippler, Bulelwa Mtukushe, Mluleki Matiwane, Kathryn E Muessig, Maryam Al-Mujtaba, T Harper Wilkinson, Alyssa Platt, Joseph R Egger, Lisa B Hightow-Weidman

**Affiliations:** 1 School of Nursing Duke University Durham, NC United States; 2 Duke Global Health Institute Duke University Durham, NC United States; 3 Department of Psychiatry and Mental Health University of Cape Town Cape Town South Africa; 4 College of Nursing Florida State University Tallahassee, FL United States; 5 Duke University Durham, NC United States

**Keywords:** HIV, adherence, mHealth, digital health, smartphone, adolescents and young adults, youths, South Africa, social support, randomized, controlled trial, pilot, antiretroviral therapy, sexually transmitted, app, apps, mobile phone

## Abstract

**Background:**

Adolescents and young adults with HIV repeatedly demonstrate low rates of antiretroviral therapy (ART) adherence as well as low rates of viral suppression. Digital health interventions are a promising way to engage adolescents and young adults with HIV to support ART adherence. However, few digital health interventions have been developed and tested with adolescents and young adults in countries like South Africa, where the HIV burden among adolescents and young adults is greatest. *Masakhane Siphucule Impilo Yethu* (MASI; Xhosa for “Let's empower each other and improve our health”) is a comprehensive ART adherence-supporting app for South African adolescents and young adults with HIV. It was culturally adapted using the HealthMpowerment platform.

**Objective:**

The aim of this paper is to describe the protocol for a pilot randomized controlled trial examining the feasibility, acceptability, and preliminary efficacy of MASI on self-reported ART adherence and social support.

**Methods:**

We will enroll 50 adolescents and young adults with HIV ages 15-21 years. Participants will be recruited from public ART clinics linked to a large government-funded teaching hospital in Cape Town, South Africa. Participants will be randomized 1:1 into either the intervention arm receiving a full version of MASI or the control arm receiving an information-only version of the app (n=25 per arm). Participants will be asked to engage with MASI daily for 6 months. All participants will complete baseline and follow-up assessments at 3 and 6 months.

**Results:**

Study screening began in May 2022 and the first participant was enrolled on June 21, 2022. As of June 12, 2023, 81 participants have completed screeners, and 36 eligible participants have been enrolled in the pilot randomized controlled trial. Recruitment is anticipated to last through August 31, 2023, with study activities anticipated through February 29, 2024.

**Conclusions:**

There is an urgent need for innovative interventions to improve ART adherence among adolescents and young adults in settings like South Africa. If found to be feasible and acceptable, MASI could be implemented with adolescents and young adults with HIV in other parts of the country.

**Trial Registration:**

ClinicalTrials.gov NCT04661878; https://clinicaltrials.gov/ct2/show/study/NCT04661878

**International Registered Report Identifier (IRRID):**

DERR1-10.2196/47137

## Introduction

### Background

Adolescents and young adults with HIV represent a large and growing proportion of people living with HIV globally [1]. Despite considerable progress in prevention and treatment, adolescents and young adults lag behind other age groups with respect to UNAID’s 95-95-95 targets for testing and treatment outcomes [2,3]. Consistent adherence to effective antiretroviral therapy (ART) is needed to suppress HIV viral load to undetectable levels, resulting in improved health outcomes and eliminating transmission [4]. Unfortunately, adolescents and young adults repeatedly demonstrate low rates of ART adherence as well as low rates of viral suppression, with estimates of viral suppression ranging from 47% to 59% [3,5]. Adolescents and young adults with HIV face a variety of barriers to ART adherence, including patient-level factors (eg, stigma, misconceptions, secrecy, treatment fatigue, forgetfulness, and lack of self-efficacy), caregiver-related factors (lack of assistance or support), and health system-related factors (eg, inconvenient clinic times) [6]. Given their developmental stage, adolescents are particularly sensitive to contextual influences, such as a lack of social support, and experience greater distress due to social exclusion [7,8].

Over 70% of adolescents and young adults with HIV live in Eastern and Southern Africa, with South Africa accounting for 22% of all adolescents and young adults with HIV globally [1]. Despite having the greatest HIV burden, interventions designed to support ART adherence and care engagement among adolescents and young adults in settings like South Africa remain limited [9-11]. A systematic review of interventions to improve adherence among adolescents and young adults conducted in 2015 identified no studies implemented in Eastern or Southern Africa [9]. A more recent review of interventions published between 2015 and 2019 identified 7 studies conducted in Eastern or Southern Africa [12]. While this is an encouraging increase, and additional trials are underway [13-15], there remains an urgent need for innovative interventions to improve ART adherence within this important population.

Digital health interventions are a promising way to engage adolescents and young adults with HIV to support ART adherence, given their increasing access to and interest in mobile technology [16,17]. Digital health interventions can be used to target multiple factors influencing a behavior (eg, knowledge, attitudes, skills, social support, and norms), which makes them well-suited to address a complex behavior like ART adherence. They can also be used to support web-based communities with the potential to reduce social isolation and loneliness while providing participants with emotional and informational social support [18,19]. Mobile phone access in South Africa was 95% as of 2018, and an estimated 91% of all phones in the country are smartphones [20,21]. Data from South Africa show that smartphone ownership is higher among young people [22], who consistently express more interest in adopting technology, making digital health interventions targeting adolescents and young adults feasible and potentially scalable in this setting.

Despite their promise, few digital health interventions to date have been developed and tested with adolescents and young adults in Eastern or Southern Africa. Among those that have been evaluated, basic applications such as SMS text messaging and phone calls have been the most common [23]. The findings of such SMS text messaging–delivered interventions have been mixed [24], highlighting the limitations of addressing only 1 adherence challenge (eg, forgetfulness), and underscoring the potential of incorporating multiple behavior change techniques (BCTs) to comprehensively address the varied adherence barriers experienced by adolescents and young adults.

To address these gaps, we developed *Masakhane Siphucule Impilo Yethu* (MASI; Xhosa for “Let’s empower each other and improve our health”), an ART adherence-supporting app for South African adolescents and young adults with HIV, based on formative research in Cape Town, South Africa [25]. MASI was culturally adapted using our team’s evidence-based HealthMpowerment (HMP) platform. HMP was built upon years of human-centered design and research [26-29] and has demonstrated improvements in HIV risk behaviors, HIV care engagement, and stigma among youths in the United States [29,30]. The HMP platform is built to support use in low-resource settings like South Africa; it includes offline support to allow use without internet access and allows participants to customize data use, which is important when wireless coverage is limited and data use fees apply.

### Objectives

The goal of this study is to conduct a pilot randomized controlled trial (RCT) of MASI with 50 adolescents and young adults with HIV, ages 15-21 years, to (1) assess its feasibility and acceptability and (2) explore preliminary effects on ART adherence and social support. This study’s protocol is guided by the SPIRIT (Standard Protocol Items: Recommendations for Interventional Trials) guidelines [31], and the CONSORT (Consolidated Standards of Reporting Trials) reporting guidelines for pilot and feasibility trials [32].

### MASI Intervention

#### MASI Mobile App Overview

MASI is a comprehensive intervention designed to promote ART adherence and foster social support using a strengths-based approach. MASI was adapted through formative research with South African adolescents and young adults with HIV, including in-depth interviews with 15 adolescents and subsequent beta testing with 12 adolescents [25]. The interviews, conducted between January and March 2021, addressed participants’ health-related challenges and unmet needs to inform the adaptation process and the development of tailored content to meet their needs. The majority of participants also described their preference for having content in English rather than Xhosa, the predominant home language of our study participants, noting the simplicity of English and the increased comfort with the language among youths [25].

After adaptation, MASI was beta tested with 12 adolescents with HIV between September and December 2021. Beta testing supported the feasibility of using MASI on participants’ devices and identified no major technical issues [33]. Our mixed methods analysis of participant paradata (ie, back-end app use metrics [34]) and in-depth interview transcripts after beta testing found MASI to be functional, usable, and highly desirable [35]. Modifications to MASI were made prior to the pilot trial based on findings from beta testing, including the addition of new resources and activities, modifications to the app programing to promote ease of navigation, and a plan for posting new content in the app at the start of the pilot RCT to encourage participant engagement (eg, icebreakers and polls).

**Figure 1:**
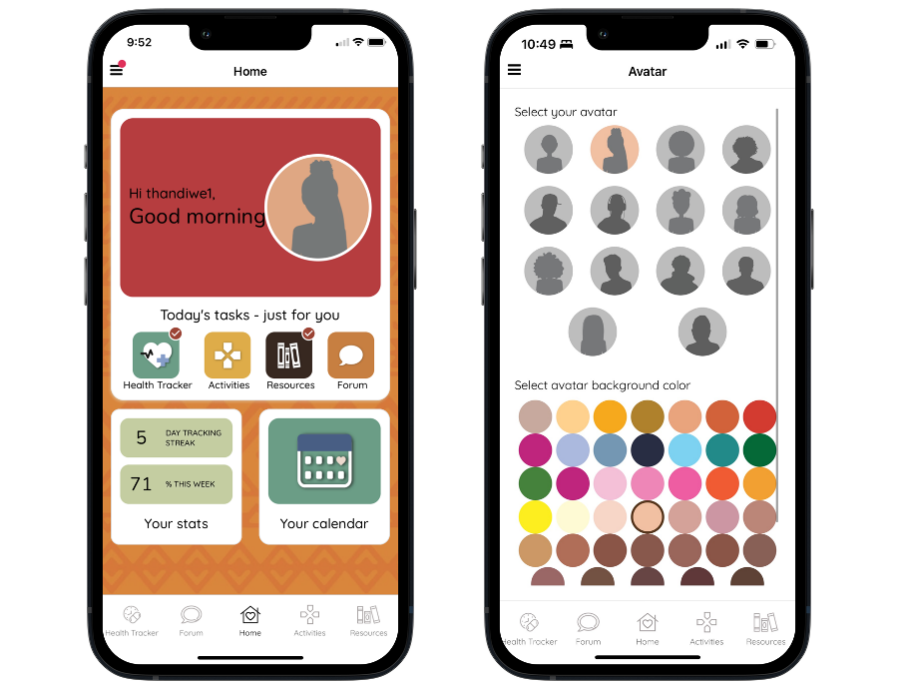
*Masakhane Siphucule Impilo Yethu* home screen and avatar customization options.

The MASI mobile app is available for Android and iOS and smartphones from either Google Play or the Apple App Store; access to the app requires a code provided by the research study. Users select a username and password and are encouraged to pick a username that does not contain any identifying information so that they can remain anonymous to other app users. Users also customize an avatar, which is displayed on the home page, along with time-specific greetings (Figure 1). The home page also includes a list of “Today’s tasks” related to 4 of the main app features: Health Tracker, Activities, Resources, and Forum. The Activities and Resources button takes participants to a prespecified activity or resource for the day. Once completed, the home screen displays a red check mark on the feature’s button in the “Today’s tasks” section.

#### MASI Mobile App Features

Core MASI features are described in Table 1, with their associated BCTs. The BCTs were identified using a taxonomy developed by Michie et al [36], which classified 93 hierarchically clustered techniques (clustered within 16 groups).

**Figure 2:**
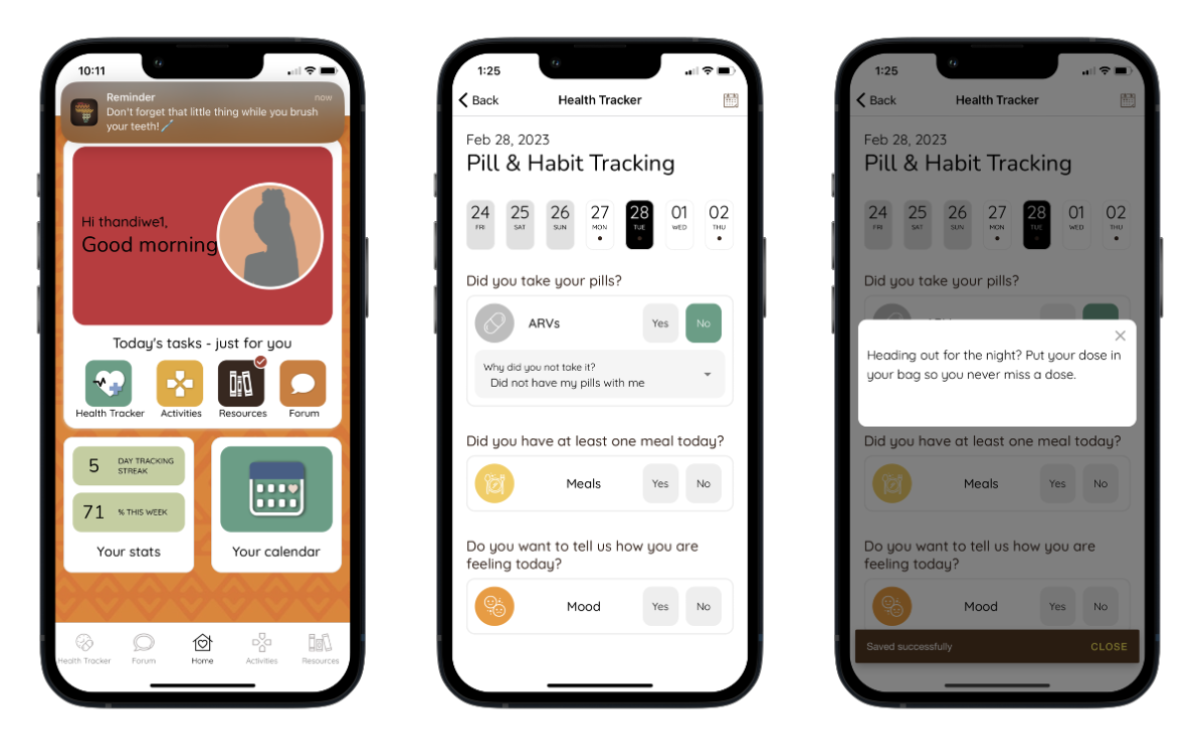
Home screen displaying reminder push notification and Health Tracker, highlighting feedback provided on antiretroviral therapy adherence behavior.

The MASI Health Tracker (Figure 2) prompts users to add details about their HIV medications (eg, frequency and time of day), and then allows users to pick a customized, tailored reminder to be sent by the app as a push notification at the selected time. If a user clicks on the reminder, they can track their adherence. If a user indicates that they missed a pill, they are prompted to provide a reason, and the app provides tips on how to overcome that challenge in the future. Users can also track other health behaviors and statistics, including meals, mood, and alcohol consumption. They can view graphs and visuals of their tracked health behaviors over time.

**Figure 3:**
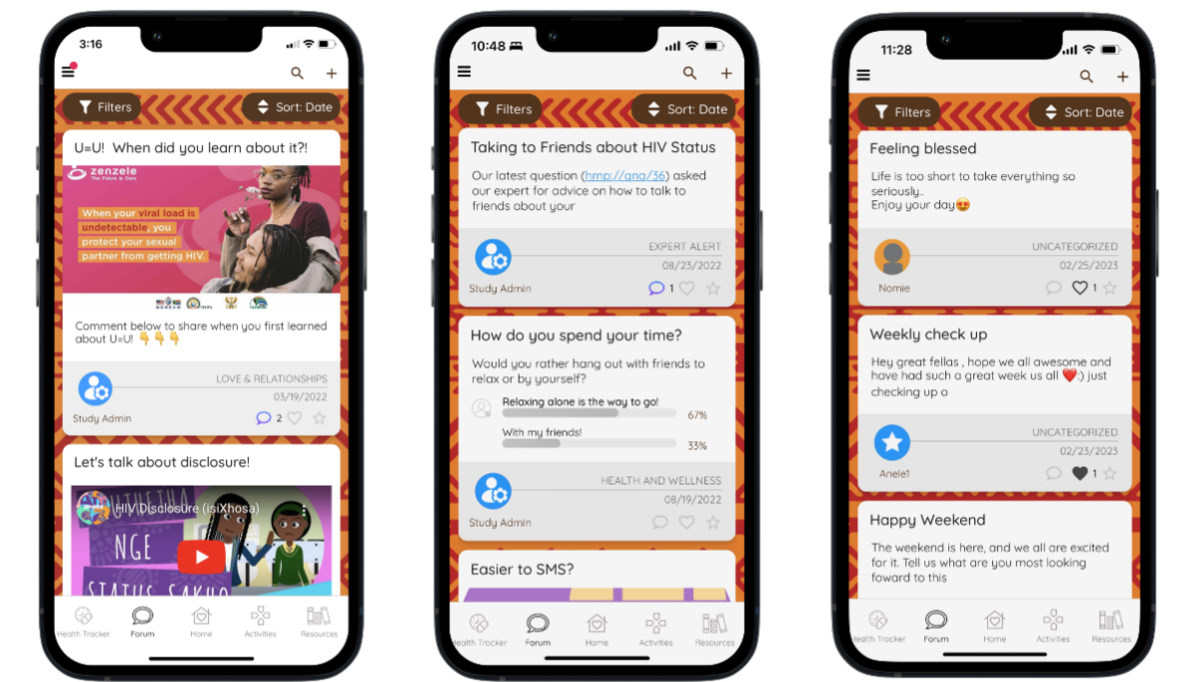
Forum displaying images, YouTube videos, polls created by study staff, and posts by study participants and peer mentors.

The MASI Forum (Figure 3) allows participants to interact with other MASI users and peer mentors by starting or adding to existing discussion posts. Users can upload images, videos, or links and can favorite posts and follow other users. Research staff, including peer mentors from the clinic who have been hired to support our MASI users, monitor and add to forum posts or create their own icebreaker posts to encourage dialogue.

**Figure 4:**
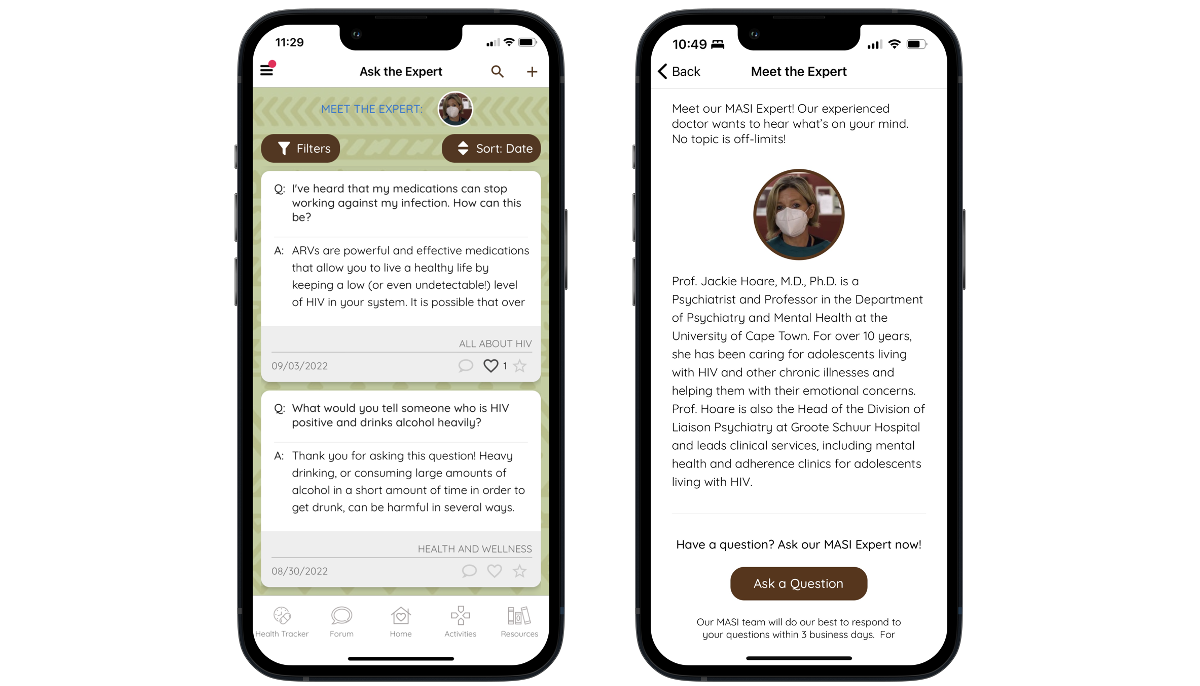
Ask the Expert feature, displaying submitted questions and preview of answer.

The Ask the Expert feature (Figure 4) allows users to submit questions that are answered by a doctor or counselor, sourcing credible information. Questions are posted anonymously, and responses are visible to all participants. Responses from the expert often link to further resources within the app related to the topic of interest.

**Figure 5:**
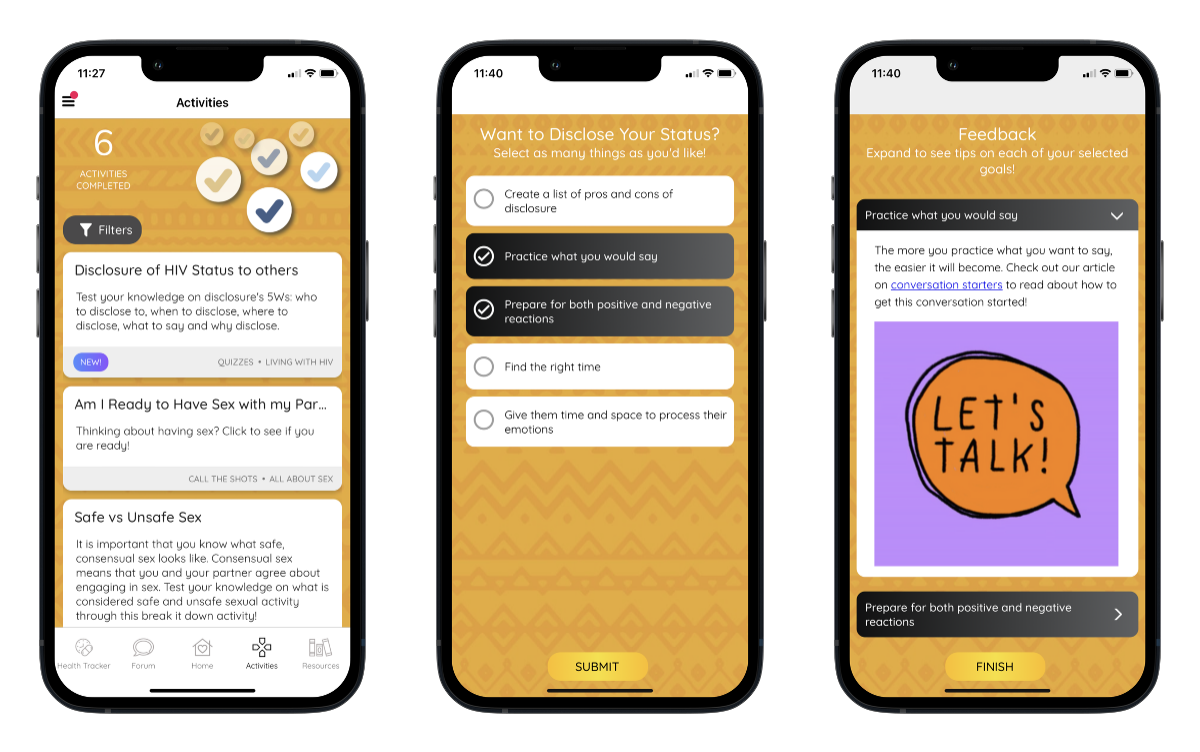
Activities landing page and example of disclosure goal-setting activity.

The Activities feature (Figure 5) offers engaging activities covering a range of health-related and lifestyle topics. Example activities include quizzes that test knowledge on a subject and provide instant feedback and explanations of the answers. Goal-setting activities allow for the users to identify action steps they want to take toward a goal and receive tips on improving selected behaviors. Other activity types include self-assessments or fill-it-in activities that allow for users to reflect on a prompt and enter in responses, such as identifying motivations for staying in HIV care.

**Figure 6:**
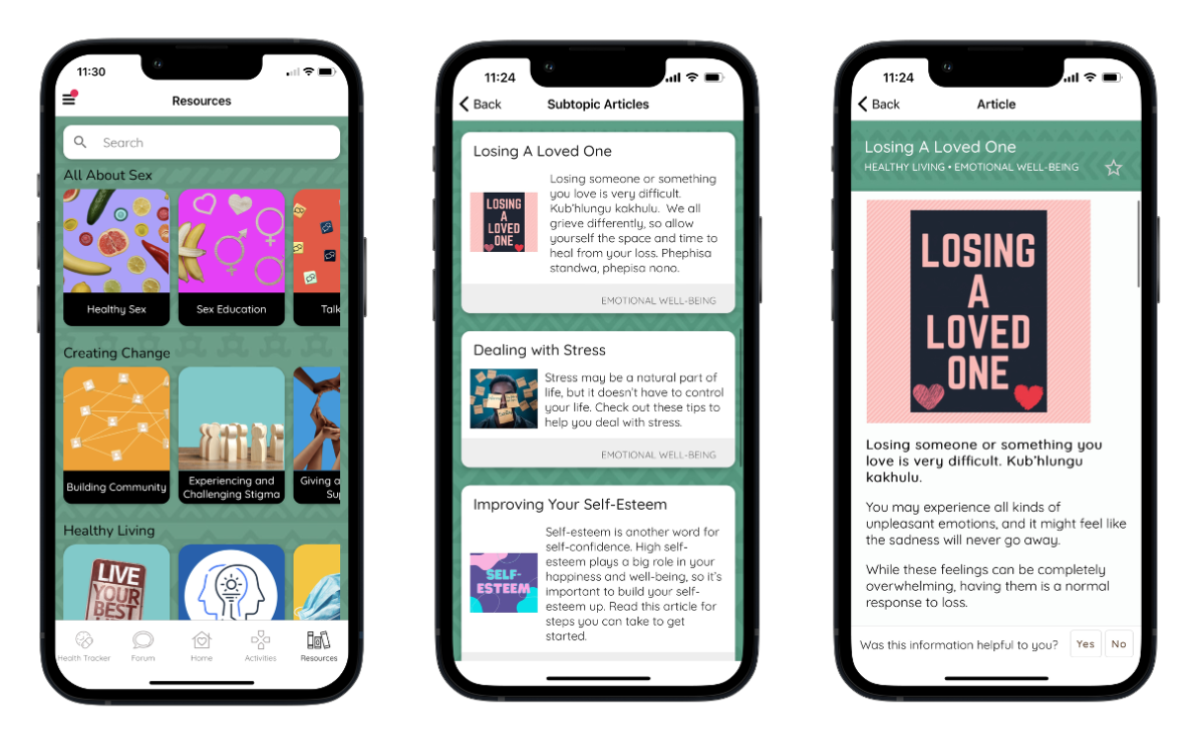
Resources landing page, example of articles within a subtopic, and example article.

The MASI Resource feature (Figure 6) provides access to multimedia resources and information on various health topics. There are over 140 articles and videos, with new content added to the resource center as it is developed. All content has been developed or adapted to the South African setting, including links to local websites or resources for more information and support. Resource topics include healthy living, love and relationships, living with HIV, all about sex, life skills, prevention, and creating change. Many of the resources contain links to other articles related to the topics, or to a relevant activity, such as a quiz to test their knowledge or a goal-setting activity.

**Figure 7:**
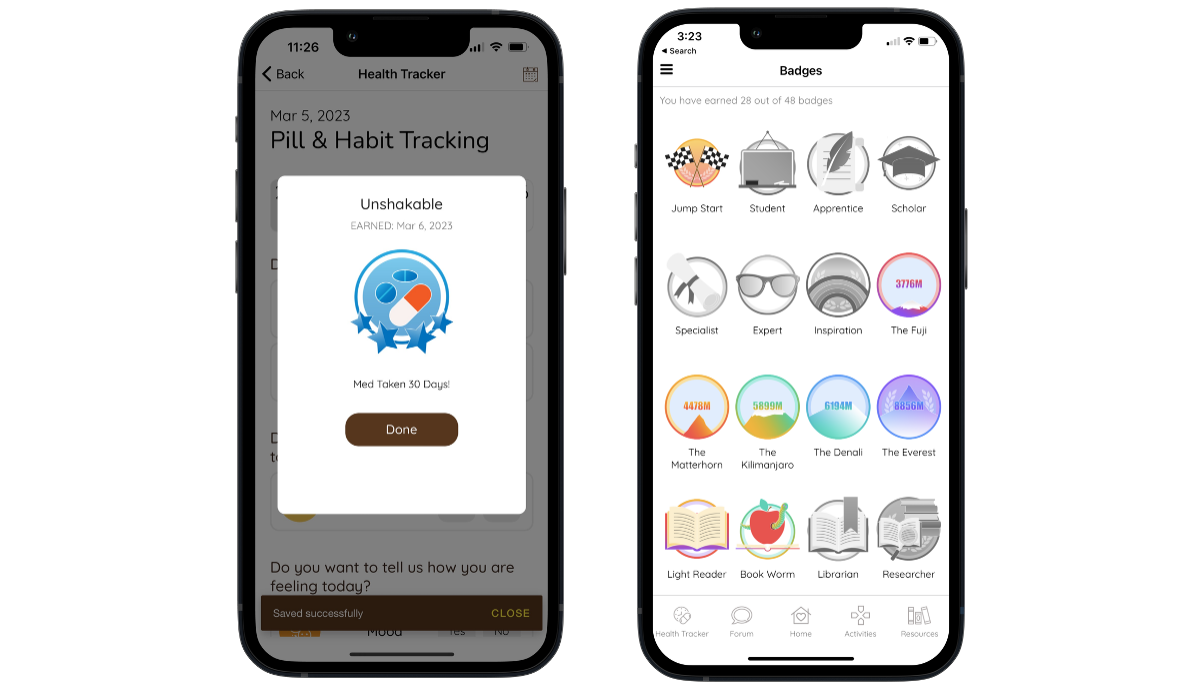
Notification for receipt of “Unshakable” badge and screen of all badges.

The MASI app also includes elements of gamification, such as badges (Figure 7) that are awarded to participants based on their app use. For example, users are awarded the “Book Worm” badge after reading 10 articles and earn the “Unshakable” badge once they report that they have taken their medication 30 times.

**Table 1:** MASI^a^ core features and associated behavior change techniques.

MASI feature	Description of feature	Behavior change techniques [36]
Health Tracker	Tracking available for ART^b^ adherence and other health-related behaviors and statistics, including meals, mood, financial stress, smoking, alcohol, drugs, sex life, CD4^c^ count, and viral load. Customizable reminders sent via notifications, with recommended text generated to connect taking medication with the timing of other daily behaviors (eg, brushing teeth). Feedback based on entered data to encourage behavior change.	Prompts or cues Feedback on behavior Self-monitoring of behavior Self-monitoring of outcomes of behavior Habit formation
Forum	Forum threads can include images, YouTube videos, or text. Users can start or contribute to existing forum threads or “like” or “favorite” posts. MASI peer mentors and staff monitor the forum and add posts, including polls, to boost engagement.	Social support (unspecified) Social support (emotional) Role-modeling Social comparison
Resources	Multimedia resources and information on health and wellness, stigma, resilience, and life skills. Information about consequences of various HIV-related behaviors (eg, disclosure and ART adherence). Resources inclusive of various learning styles and health literacy levels. Resource articles link to care locator with up-to-date local resources available.	Instruction on how to perform behavior Information about health consequences
Activities	Activities include risk assessments, quizzes, goal setting, sorting, and matching.	Goal setting (behavior) Goal setting (outcome)
Ask the Expert	Direct connection to a health care provider who answers questions submitted within the app.	Credible source
Game-based elements	Badges awarded for prespecified targets (eg, 30 min using MASI). Avatars represent MASI users and can be swapped out for various colors and silhouettes.	

^a^MASI: *Masakhane Siphucule Impilo Yethu*.

^b^ART: antiretroviral therapy.

^c^CD4: cluster of differentiation 4.

#### MASI Peer Mentors

Our study will hire at least 2 part-time MASI peer mentors to provide support to MASI users. Peer mentors will be between the ages of 18 and 29 years with a track record of good adherence to ART and HIV care engagement, strong communication skills, and demonstrated leadership qualities. We will hire individuals who have been trained to serve as in-person peer mentors and facilitate in-person support groups at local ART clinics. MASI peer mentors will be trained on how to use the app, and they will be asked to interact with MASI intervention participants through the forum feature. Their role will be to post icebreakers, respond or react to participant posts, share their own personal experiences to relate to participants, and help generate ideas for new content.

#### MASI Content Library

The MASI Content Library is a compilation of the materials shared on the MASI app via the administrative portal, including multimedia resources and activities, “Ask the Expert” questions and responses, and forum posts. It also includes materials to support this content, such as a catalog of images and GIFs to be used in forum posts, articles, and activities.

Content is developed and curated from multiple sources, including the HMP Content Library and web-based resources designed for South African adolescents and young adults (eg, Amaze animated videos on sexual health topics adapted by Marie Stopes International for use in South Africa [37]). All content is adapted and reviewed by the South African research team to ensure cultural relevance, age appropriateness, and accuracy of listed local resources (eg, where to access pre-exposure prophylaxis locally).

The initial content available on the app at the start of the pilot RCT covers a wide variety of topics related to HIV, general health, safe sex, and lifestyle. Additional content will be generated and added to the app in response to participant interests, input from peer mentors, and emerging topics in the news. Topics for additional content will be identified by reviewing Forum posts and Ask the Expert questions for topics of interest, examining the app-generated paradata for content searches by users, and asking participants for feedback during data collection. This process was modeled during our beta testing phase, during which resources related to sexual and gender diversity were developed and added to MASI in response to a participant’s searches for resources and a question to experts about same-gender-loving relationships.

#### MASI Admin Portal

The MASI administrative (admin) portal is a web platform that allows study administrators to manage the content on the MASI app, manage participant access to the app, and review paradata generated by participants’ app use. All content that is live on the app is housed in the admin portal, which allows for links across features and content (eg, links within an article to additional resources or related activities). When a participant asks a question to the expert on the app, the response can be drafted and posted within the admin portal to appear on the app, and this study’s team can add a forum post alerting participants of a new question. The admin portal allows for scheduling of posts so that content can be developed in advance and released at ideal times. This study’s team can also manage notifications that participants receive in-app or via SMS text messaging, which includes notifications about new articles, responses to participant forum posts or messages, or encouragement to log in if users have been away from the app for a few days.

The data analytics feature of the admin portal allows this study’s team to run ad hoc queries examining the paradata for quick feedback on app use [34,38]. User data will help inform the development of new content tailored to user’s interests or preferences by identifying gaps in content or topics and activity types with a high level of user engagement.

## Methods

### Study Design

This study is a pilot RCT that will enroll 50 adolescents and young adults with HIV. Participants will be stratified by self-reported sex assigned at birth and randomized 1:1 (n=25 per arm) into 2 parallel arms: the intervention arm receiving a full version of MASI or the control arm receiving an information-only version of the app. Participants will be asked to engage with MASI daily for 6 months.

### Study Setting

This study takes place in Cape Town, South Africa. In South Africa, HIV prevalence is significantly higher among Black Africans compared to other racial groups. These racial disparities are rooted in structural and contextual inequalities, including the legacy of apartheid policies that produced economic and health advances for those classified as White [39].

Participants will be recruited from public ART clinics linked to a large tertiary government-funded teaching hospital in Cape Town. As a tertiary hospital, it offers a standard of care to all patients, who are admitted to the facility through referrals from primary and secondary health care facilities. In addition, it offers specialized pediatric and adolescent ART services with a qualified and experienced team of clinicians such as psychiatrists, psychologists, nurses, and HIV lay counselors.

### Participants

Participants will be eligible for this study if they meet the following eligibility criteria: (1) they are aged ≥15 years and ≤21 years, (2) they know their HIV status (screened participants who do not know their HIV status will receive information on free voluntary HIV counseling and testing services), (3) they are living with HIV, (4) they have been prescribed medication to treat HIV, (5) they are not attending school for learners with special needs (eg, School of Skills) and have not repeated a grade in school more than once (this is to increase the likelihood that adolescents have the decisional capacity to provide informed consent to participate in this study and to reduce the risk of the research activities causing confusion among participants with diminished capacity or cognitive impairment), (6) they have a smartphone that can download apps, (7) they feel comfortable using an app with content in English, (8) they have no plan to move outside of Cape Town in the next 6 months, (9) they have not previously participated in the MASI beta testing phase of our study, and (10) they are able to successfully install the MASI app on their smartphone.

### Procedures

#### Recruitment and Screening

Participants will be recruited at ART clinics in Cape Town, South Africa. Peer mentors will work with clinic nurses to identify potential participants in clinic waiting rooms to share details about this study, assess their interest in learning more, and collect their contact info for screening. We will also recruit participants from other research studies who have provided consent to be contacted for future research.

Potential participants will be screened by phone after providing verbal consent to see if they meet the eligibility criteria for this study. Participants who meet these criteria will be invited for an in-person appointment. After completing the informed consent or assent process (including consent from an adult caregiver for participants between 15 and 17 years of age), this study’s team will confirm that the participant is able to successfully install the MASI app on their smartphone. Screening data will be entered electronically by the interviewer using Research Electronic Data Capture (REDCap), a secure, web-based application hosted at Duke University [40]. REDCap will also be used to track the status of potential participants, including documenting reasons for declining participation and ineligibility.

#### Baseline Data Collection

Eligible participants will complete a baseline assessment, an interviewer-administered survey with data entered electronically by the interviewer using REDCap. REDCap provides an intuitive interface for validated data entry, audit trails for tracking data manipulation and exporting, and automated export procedures for data downloads to common statistical packages. For survey questions with a higher potential for social desirability bias (eg, sexual behavior and study feedback), participants will be offered the opportunity to self-enter data into REDCap. The assessment items will be displayed on the screen in both English and Xhosa, the most commonly spoken home language among our study participants [33], and assessments will be conducted by bilingual interviewers.

#### Randomization

After completing the baseline assessment questions, participants will be randomized at a 1:1 ratio to the intervention condition (full MASI app) or control condition (information-only version of MASI). In total, 25 participants will be assigned to each condition and participants will be stratified by self-reported sex at birth. Randomization will be revealed by this study’s interviewer using the REDCap Randomization Module, which uses a computer-generated allocation sequence created in Excel and uploaded to the module prior to the start of the trial. This study’s interviewers will be unaware of this study condition prior to allocation. Participants will not be blinded to their allocation, as all participants will become aware of the full MASI features during the informed consent process.

#### Experimental Conditions

##### Intervention

Participants randomized to the intervention condition will receive access to the full MASI app, including all its features (ie, Health Tracker, Forum, Ask the Expert, Activities, and Resources). Through the Forum feature, participants will be able to interact anonymously with the other intervention participants, along with peer mentors and study staff.

This intervention is intended to supplement existing clinical care, so it will not impact access to routine medical care.

##### Control

**Figure 8:**
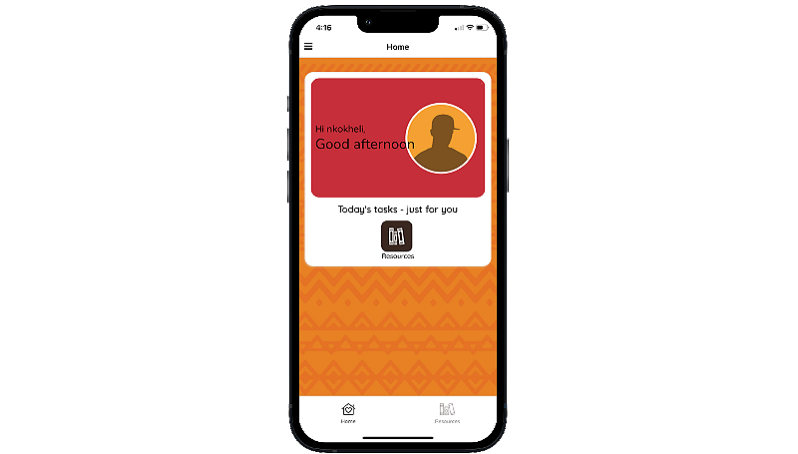
Home screen for users in the information-only control group.

Participants randomized to the control condition will receive access to an information-only version of MASI. Control participants will have access only to the Resources feature (Figure 8). An information-only version of MASI was chosen as the comparator given recommendations from the local study team, the need to download MASI to confirm the final eligibility requirement of this study, and the benefit of equalizing contact with participants to check-in on app access and provide smartphone data.

#### Onboarding

Once participants have been allocated to their condition, a research assistant will help them set up their profile and username. Participants will be advised to pick a username that does not include their real name, email address, or other social media account names. The research assistant will then walk the participant through the MASI features and demonstrate how to navigate the app and how to contact this study’s staff if needed. Participants will be asked to log into the app daily and will be given 1 GB of smartphone data each month to support their app use.

#### Follow-Up Assessments

Participants will complete assessments at 3 and 6 months after enrollment; follow-up assessments will aim to be scheduled as close to the 3 or 6 month date as possible but may occur within 4 weeks before or after the target dates to accommodate participant scheduling (eg, holidays and school breaks). Follow-up assessments will be administered in the same way as the baseline assessment, using REDCap data collection software. Assessments will include a few open-ended questions to give participants the opportunity to share feedback on things they liked about MASI, note any challenges experienced, or explain periods of lost access to the app (eg, lost phone and forgot password). In the event that it is not possible to conduct in-person data collection, follow-up assessments prioritizing main study outcomes may be conducted by phone.

#### Participant Tracking and Retention

Research staff will inquire about preferred and acceptable methods of communication, including alternative contacts if participants cannot be reached on a primary phone number. Participants will be contacted each month to send a 1-GB data top-up to support their app use. At this time, study staff will confirm if the participant still has access to their phone and the MASI app and will document any shared reasons for app nonuse. Study staff will also help troubleshoot any app-related technical challenges encountered.

To promote participant retention, research staff will emphasize the importance of attending all assessment appointments. Ahead of the 3- and 6-month study visits, research staff will arrange for a registered transportation company to collect participants (and accompanying adults, if aged 18 years or younger) in their area of residence and bring them to the research site for assessments. We will reschedule appointments as needed and will follow-up with participants who miss appointments.

#### Reimbursement

Participants will receive R250 (approximately US $14.50) at each visit. Participants (and their adult caregivers, if applicable) will also be given refreshments, including a snack and a drink.

#### App Access After Conclusion of Study

Participants will consent to 6 months of app access. After 6 months, the app will display a message informing the participant that they have completed this study and no longer have access to the app. The message will thank participants for their participation and encourage them to share any additional feedback by contacting our study team. Content generated by participants (eg, forum posts and Ask the Expert questions), will still be visible to other users on the app after their access ends. The decision to end access to the app was made due to our ethical obligations to monitor and be responsive to content from active users and the nature of on-going data generated from app use.

### Outcomes

Primary outcome measures include intervention feasibility measures recorded by app backend paradata (the number of days participants log into the app, the total time participants spend using the app, and the number of days participants log medications using the app) and intervention acceptability (mean composite score from the adapted System Usability Scale [41,42]) at 3- and 6-month follow-ups.

Secondary outcome measures include adherence to ART (30-day recall of missed doses) and perceived social support using the adapted Medical Outcomes Study Social Support Survey scale [43,44] at 3- and 6-month follow-up.

All other measures are exploratory and include HIV/AIDS knowledge and stigma. HIV/AIDS knowledge will be measured using a scale adapted from a United States Agency for International Development toolkit for transition of care for adolescents with HIV [45] and a scale measuring HIV treatment knowledge in context of treatment as prevention [46]. Stigma will also be measured with the Adolescents Living with HIV Stigma Scale, which includes internalized stigma (5 items), anticipated stigma (2 items), and enacted HIV stigma (3 items) [44,47,48]. We will also measure various psychological and behavioral covariates as well as demographic characteristics. HIV disclosure and disclosure intentions will be measured using items previously used with adolescents living with HIV [49]. Disclosure concerns will be measured using 2 items from the HIV Stigma Scale for children [50]. Anxiety will be measured with the General Anxiety Disorder scale [51]. Depression will be measured with the Patient Health Questionnaire-9 [51]. Social isolation will be measured using the National Institutes of Health Toolbox Loneliness Scale (for ages 8-17 years) [49]. Resilience will be measured using the Connor-Davidson Resilience Scale 10 [52]. Demographic information collected will include age, sex assigned at birth and current gender identity [53], population group, and home language. Participants will also self-report if they were born with HIV.

Primary and secondary outcomes are described in Textbox 1 along with potential covariates and proxy outcomes of interest. To the extent possible, outcome measures were selected based on their prior use with adolescents and young adults with HIV in South Africa. Measures without existing Xhosa translations were translated from English into Xhosa and then back-translated, with team discussion to determine the best translation for items. Items will be displayed on the REDCap data collection screen in both English and Xhosa.

**Pilot randomized controlled trial primary and secondary outcomes.**

**Primary outcomes**

- Intervention feasibility:
  Number of days participants log in to the app as recorded by app backend paradata (3 and 6 months).
  Total time participants spend using the app (minutes), as recorded by app backend paradata (3 and 6 months).
  Number of days participants log medications using the app as recorded by app backend paradata (3 and 6 months).
- Intervention acceptability:
  Average score of an adapted version of the system usability scale [41,42]. Total possible range: 0-100. Higher score indicates higher usability and helpfulness (better outcome). Average System Usability Scale score >68 (average for digital health apps [54]) will be considered acceptable (3 and 6 months).

**Secondary outcomes**

- Adherence to antiretroviral therapy:
  Self-reported number of missed antiretroviral therapy doses in the past 30 days (3 months and 6 months).
- Perceived social support:
  Adapted version of the Medical Outcomes Study Social Support Survey [43,44], which measures 4 domains of social support: emotional or informational support, tangible support, affectionate support, and positive social interaction. Total possible range: 12-48. Higher score indicates higher social support (better outcome; 3 months and 6 months).

### Statistical Analysis

This study will enroll 50 adolescents and young adults with HIV into this study (25 males and 25 females based on self-reported sex assigned at birth). This study’s sample size (N=50) was selected to be large enough to reasonably evaluate the feasibility and acceptability of MASI while balancing budgetary constraints [55].

Descriptive summaries will be computed for all study outcomes with continuous variables summarized with means and SD (or medians with IQR in the case of skewed distribution) and categorical variables summarized with counts and proportions. Outcomes will be summarized, stratified by treatment group, at each available study time point.

As a pilot study with a primary focus on feasibility and acceptability, the examination of MASI’s preliminary efficacy will be exploratory. To explore MASI’s preliminary efficacy on ART adherence and social support at 3 and 6 months, we will use linear mixed models to compare outcomes among participants randomized to MASI compared versus the information-only control at each time point. Log transformed outcomes and alternative modeling approaches such as Poisson and negative binomial will be considered in the event that assumptions for the linear model are violated. All models will include a fixed effect for sex assigned at birth (stratification variable) and a random intercept term to account for correlation in the response due to repeated measures within study participants over time. A random slope for participant will also be considered, as will the covariance structure of the errors (eg, exchangeable and autoregressive). Distribution of residuals will be examined to confirm that modeling assumptions are met. Because sample size is small (this study is not powered to detect clinically meaningful effect sizes) and the regression analyses are exploratory in nature, inference will focus on the size and direction of the total treatment effects rather than statistical significance.

Additional exploratory analyses will examine MASI’s effects over time on HIV/AIDS knowledge and stigma using methods similar to those used for adherence and social support outcomes. We will also conduct descriptive analyses examining trends and patterns in user engagement with various components of the MASI interface over time.

### Monitoring

This study’s procedures have been determined to be low-risk, and we do not anticipate any adverse events as a result of study participation. While a data monitoring committee is not needed for this study, the research team will meet regularly to report on study progress and monitor compliance to this study and ethics protocols.

Our team has developed a set of standard operating procedures (SOP) for various scenarios that includes instructions for following up in response to concerns about participant distress, child abuse, sexual assault, underage sex, violence toward others, and suicidal ideation. Study staff are trained to identify situations of concern, including monitoring posts on the MASI app and participant comments or behavior during in-person study activities. Our data collection surveys include 2 items that assess for suicidal ideation [48,56] (ie, endorsement of “thoughts that you would be better off dead or of hurting yourself in some way” and feelings that one “would rather die than live with HIV”). If either item is endorsed, a message will be displayed to the interviewer at the end of the assessment to remind them to initiate the SOP. The SOP includes appropriate follow-up steps and procedures for situations where reporting to outside authorities is required by South African law (eg, mandatory reporting of child abuse).

### Ethics Approval

This study has been approved by the ethical review boards at the University of Cape Town (Ref 600/2020) and Duke University Health System (Pro00103309). The ethical review boards will meet annually to review the trial progress and conduct throughout the trial period. The trial is registered at ClinicalTrials.gov (NCT04661878).

## Results

Study screening via phone calls began in May 2022 and the first participant was enrolled on June 21, 2022. As of June 12, 2023, 81 participants had completed screeners, and 56 participants were considered preliminarily eligible for this study. Of these, 38 attended an in-person visit to confirm eligibility (including successful installation of the MASI app) and complete the baseline survey.

In total, 36 participants have been enrolled, have completed baseline, and have been randomized to a study arm. Further, 25 participants have completed a 3-month follow-up survey (100% retention rate to date), and 15 participants have completed a 6-month follow-up survey (79% retention rate to date).

Recruitment is anticipated to last through August 31, 2023, to reach the target enrollment of 50 participants, with study activities anticipated through February 29, 2024, and results available in 2024.

## Discussion

### Principal Findings

There is an urgent need for innovative interventions to improve ART adherence among adolescents and young adults in settings like South Africa. Digital health interventions are a promising approach allowing for adaptation and customization to unique needs and interests, widespread reach and scalability, and discreet use. They also allow for the integration of multiple strategies to elicit behavior change, promote connection, and support well-being. As access to technology and device ownership becomes more widespread, particularly in low- and middle-income countries, a “new type of digital divide” is emerging, characterized by inequities in access to high-quality web-based opportunities and experiences [57]; in this setting, it is argued that the “most vulnerable youths” also have the most to gain from technological interventions, yet are often the least supported by digital interventions. MASI seeks to address this gap by creating a platform tailored to the needs of adolescents and young adults in Cape Town, South Africa.

MASI is a comprehensive adherence-promoting app adapted for the South African setting that capitalizes on opportunities presented by mobile health (mHealth) platforms. MASI includes multiple BCTs to address the various complex barriers to adherence experienced by adolescents and young adults. Other studies have found that interventions with more BCTs are more effective than those with a limited amount of BCTs [23,58]. The multiple BCTs are integrated into a variety of features within the MASI app that allows users to explore and use features that are most helpful and desirable to them. The collection and analysis of app-generated paradata, in combination with survey data, will allow our team to explore participants’ use of MASI across features and lend insight into what features or BCTs may be most effective or enjoyed by participants.

The comprehensiveness and customizability of MASI are possible because it is built using HMP, an existing platform originally built to support youths in the United States [26,28]. Building mHealth interventions on existing platforms has numerous benefits, including technological sophistication and increased potential for scalability. By building MASI using an existing platform, rather than developing a mobile app from scratch, we benefited from more rapid development, substantial cost savings, and achieved a more sophisticated technological product than we would have otherwise. The use of the HMP platform also allows for the lessons learned from this RCT to be more readily applied to other interventions using the HMP platform, or the potential adaptation of MASI to meet the needs of other populations. If found to be feasible and acceptable, MASI could be implemented with adolescents and young adults in other parts of South Africa.

### Limitations

Our team acknowledges limitations in our study design related to a small sample size and therefore limited power to establish the efficacy of MASI. However, the sample size is appropriate for a pilot study focused on feasibility and acceptability as primary outcomes. The follow-up time for this pilot RCT is also limited to 6 months, which will not allow us to establish the feasibility or acceptability of a longer-term intervention.

Although smartphone ownership is widespread among South African youths, not all adolescents and young adults with HIV will be eligible for our study, as participants will be required to have a smartphone to be eligible. Restricting this study to adolescents and young adults who have a smartphone will impact the generalizability of the findings. We also recognize that excluding adolescents and young adults without smartphones may deepen existing inequities and leave out individuals with limited access to resources and social support. Furthermore, participants may lose access to their devices during the course of this study, which may impact study outcomes. Future research is needed to examine best practices for reducing technology-related barriers to participation in the research process.

ART adherence will be analyzed using a self-reported adherence outcome. Self-reported adherence measures may be impacted by social desirability bias, which could lead to measurement error for the adherence outcome. Data from this pilot RCT will be used to inform a fully powered future trial that will also include biomarker data such as viral load.

Finally, it is possible that participants randomized to the intervention arm may discuss or show MASI to participants randomized to the control condition. We do not expect such contamination to be frequent, but we will measure contamination to better understand the extent to which it occurs.

## Abbreviations

ART: antiretroviral therapy
BCT: behavior change technique
CONSORT: Consolidated Standards of Reporting Trials
HMP: HealthMpowerment
MASI: *Masakhane Siphucule Impilo Yethu*
mHealth: mobile health
NIH: National Institutes of Health
RCT: randomized controlled trial
REDCap: Research Electronic Data Capture
SOP: standard operating procedure
SPIRIT: Standard Protocol Items: Recommendations for Interventional Trials

## Data Availability

Data sharing is not applicable to this paper as no data sets were generated or analyzed during this study.
